# A new hygropetric genus of Heptageniidae from southern China (Insecta, Ephemeroptera)

**DOI:** 10.3897/zookeys.1269.177719

**Published:** 2026-02-13

**Authors:** Muhe Deng, Xuhongyi Zheng, Dewen Gong, Changfa Zhou

**Affiliations:** 1 College of Life Sciences, Nanjing Normal University, 1 Wenyuan Road, Nanjing 210023, China College of Life Sciences, Nanjing Normal University Nanjing China

**Keywords:** Adaptation, habitat, mayfly, scraper, taxonomy

## Abstract

A small waterfall in Yunnan Province, China, was sampled for heptageniid nymphs and an unknown species was found, which is characterised by having expanded femora and short tibiae, tarsi, and lamellae of gills I. Furthermore, the reared male imagos have longer hindtarsi than tibiae and almost totally fused penes. Based on these key characters and the hygropetric particularity of the new species, a new genus is established in the subfamily Ecdyonurinae. Molecular and morphological evidence demonstrate *Brachypodus quadratus***gen. et sp. nov**. is closely related to *Thalerosphyrus* and *Atopopus*. Two modified keys to common Oriental genera in the subfamily Ecdyonurinae are also provided.

## Introduction

Heptageniidae is the third largest family within the order Ephemeroptera, with more than 600 species described worldwide (Jacobus et al. 2019). Although the heptageniids of Europe and North America have been well studied, those of the Oriental region and South America remain poorly understood, especially the species belonging to the subfamily Ecdyonurinae.

A comprehensive collection and study are currently underway in China. In our mayfly collection, most species cannot be definitively assigned to a proper genus. In recent years, a series of genera within this family have been established (*Parafronurus* Zhou & Braasch, 2003; *Regulaneuria* Zhou, 2021; *Maculogenia* Zhou, 2022), demonstrating that the diversity of heptageniids in Asia is far greater than previously recognised (Zhou and Braasch 2003; Lei et al. 2021; Gong et al. 2022).

Most heptageniids are typical scrapers that inhabit stones. Some species, however, are adapted to extreme habitats, such as waterfalls, alpine creeks, and rock surfaces in running currents. Unfortunately, these specific areas and microhabitats have not been thoroughly surveyed in China.

During the spring of 2025, we attempted to collect mayflies from a waterfall in Yunnan Province, southwest China. Surprisingly, a great number of heptageniid nymphs were discovered there. Subsequent rearing provided us with more than ten adults. After careful examination, we determined that the medial depression of mesothoracic furcasternum divergent anteriorly in imagos confirms that the species is from Ecdyonurinae (Webb and McCafferty 2008). However, the combination of relatively short legs and other diagnostic characteristics the nymphs possess distinguishes them from all the known genera in this subfamily. Therefore, a new genus is proposed, and its differences from other related genera are provided and discussed in detail.

## Materials and methods

The nymphs were collected using a hand net, and mature individuals were reared outdoors until emergence. All materials were stored in ethanol (85%). The specimens used in this study are deposited in the Mayfly Collection, College of Life Sciences, Nanjing Normal University (**NJNUMayfly**).

All specimens were examined using a stereomicroscope (Nikon SMZ 745T) and photographed with a SONY a7R II camera equipped with a LAOWA 25 mm 5× macro lens. Some small structures, like claws, spatulate setae on femora and gills, were photographed using a camera (Nikon 50i) mounted on a microscope.

Samples for scanning electron microscopy (SEM) were prepared according to a standard protocol: dehydrated through an acetone gradient (70%, 80%, 90%, 98%, 100%, 10–20 min each), followed by treatment with 1–1.5 mL hexamethyldisilazane (HMDS) for 30 min. The samples were then left in a vacuum container for desiccation overnight before being sputter-coated with a gold film prior to imaging. Eggs were dissected from female imagos.

The DNA extraction and amplification followed the process by Zheng and Zhou (2021). The COI sequences of species from other genera were downloaded from GenBank, and specimen information is listed in Table 1. All the sequences were aligned using MUSCLE, and the genetic distance was estimated using the Kimura 2-parameter model (K2P) in MEGA11.

**Table 1.** GenBank ID of sequences used in this study.

Species	GenBank ID	Species	GenBank ID
*Afronurus levis*	MN608818	*Compsoneuria* sp.2	HE651373
*Afronurus* sp.	KT250125	*Ecdyonurus insignis*	PP403787
*Asionurus* sp.1	HQ979421	*Ecdyonurus venosus*	PP403777
*Asionurus* sp.2	HM417034	*Parafronurus youi*	MH183172
*Atopopus tarsalis*	HE651380	*Thalerosphyrus flowersi*	KX376969
*Atopopus* sp.	KT250134	*Thalerosphyrus vietnamensis*	MH109094
*Compsoneuria* sp.1	HF536607	*Brachypodus quadratus*	PX056341

## Taxonomy

### Brachypodus gen. nov.

Taxon classificationAnimaliaEphemeropteraHeptageniidae

E4FED922-E7DC-505B-AE8E-BD440773810A

https://zoobank.org/DAF9D08E-DA0B-4A57-92D4-D892A9FD041B

#### Type species.

*Brachypodus quadratus* sp. nov., China (Yunnan Province).

#### Etymology.

*Brachypodus* is a combination of Latin words *brachy*- (short) and *poda* (leg), indicating the relatively short nymphal tibiae and tarsi of the type species. The specific name quadratus means the subquadrate shape of fore- and midfemora of nymphs.

#### Description.

**Figure 1.**
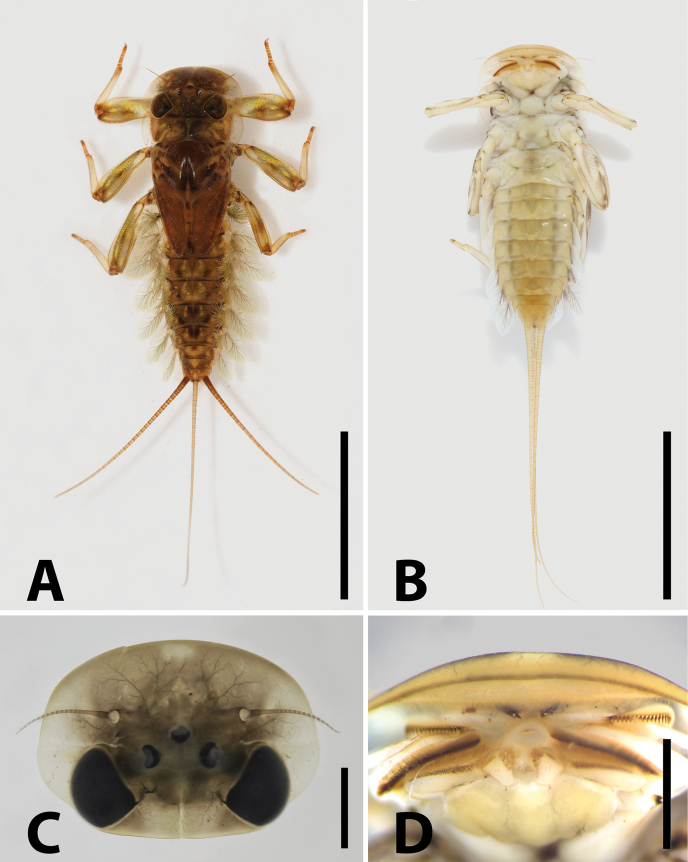
Nymph of *Brachypodus quadratus* gen. et sp. nov. **A**. Nymphal habitus, dorsal; **B**. Nymphal habitus, ventral; **C**. Head capsule; **D**. Anterior margin of head capsule (ventral view). Scale bars: 0.5 cm (**A, B**); 1.0 mm (**C, D**).

**Figure 2.**
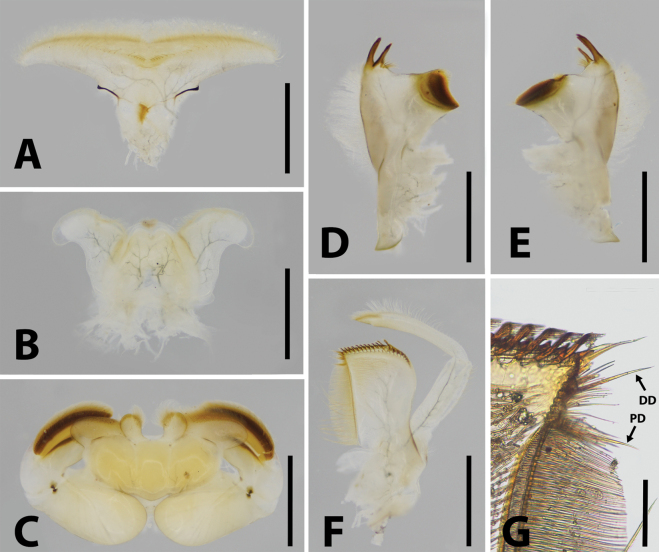
Mouthparts of *Brachypodus quadratus* gen. et sp. nov. **A**. Labrum (ventral view); **B**. Hypopharynx (ventral view); **C**. Labium (ventral view); **D**. Left mandible; **E**. Right mandible; **F**. Maxilla; **G**. Dentisetae enlarged (DD = distal dentiseta, PD = proximal dentiseta). Scale bars: 1.0 mm (**A–F**); 0.1 mm (**G**).

**Figure 3.**
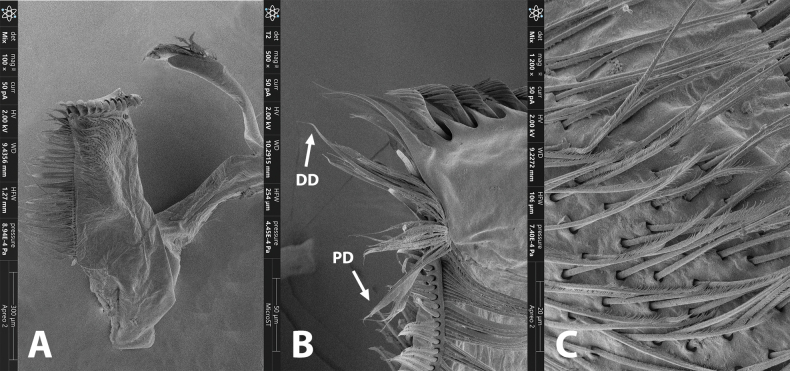
Maxilla of *Brachypodus quadratus* gen. et sp. nov. (SEM photo). **A**. maxilla; **B**. Dentisetae enlarged (DD = distal dentiseta, PD = proximal dentiseta); **C**. enlarged scattered setae on ventral surface.

**Figure 4.**
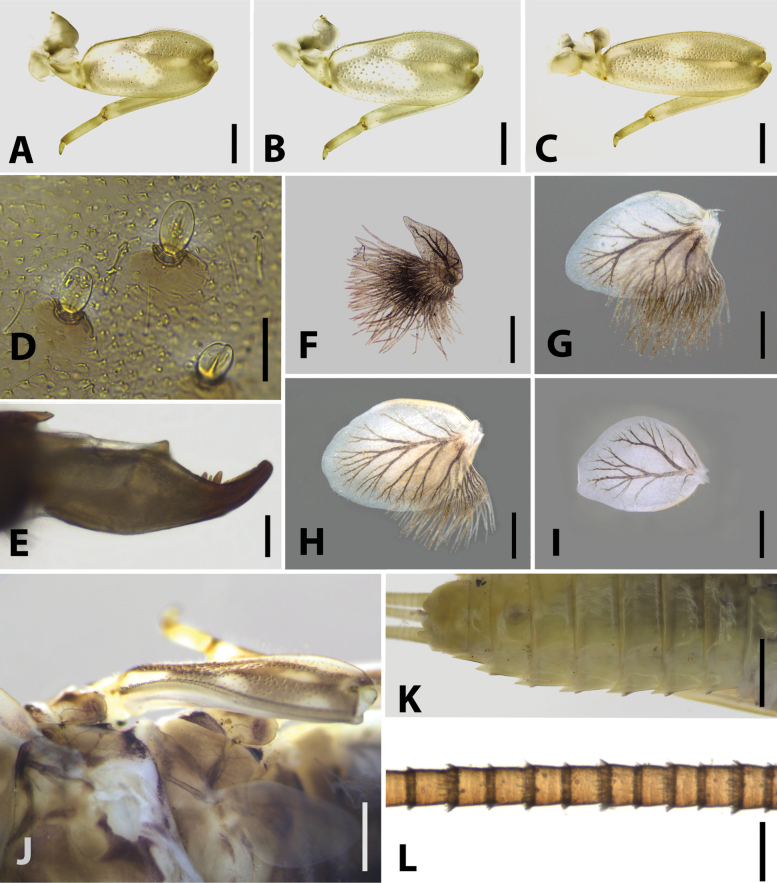
Nymphal structures of *Brachypodus quadratus* gen. et sp. nov. **A**. Foreleg; **B**. Midleg; **C**. Hindleg; **D**. Spatulate setae on dorsal surface of forefemur; **E**. Midleg claw; **F**. Gill I; **G**. Gill II; **H**. Gill VI; **I**. Gill VII; **J**. Base of mid- and hindcoxae; **K**. abdomen (ventral view, showing projected posterolateral angles); **L**. Caudal filaments (near base). Scale bars: 0.5 mm (**A–C**); 0.05 mm (**D, E**); 0.25 mm (**F–I**); 0.5 mm (**J**); 1.0 mm (**K**); 0.2 mm (**L**).

Nymph: body length around 10.0 mm, pronotum extended laterally into distinct semicircular lobes (Fig. 1A, B); posterior margin of head capsule straight, anterior margin of head capsule not thickened, with tiny hair-like setae (Fig. 1C, D); length of labrum ca 3.0× its width (Fig. 2A); superlinguae of hypopharynx extended into round lobe (Fig. 2B); mouthparts light coloured (Fig. 2A–G); distal dentiseta of maxillae single, proximal dentiseta distally bifurcate (Fig. 3A, B); ventral surface of galea-lacinia with scattered simple hair-like setae (Fig. 3C); femora of fore- and midlegs expanded and nearly quadrate-shaped, tibiae of all legs distinctly shorter than femora; combined length of tibiae and tarsi in foreleg and midleg slightly longer than femora, combined length of tibiae and tarsi in hindleg shorter than femora (Fig. 4A–C); transparent oval spatulate setae on dorsal face of femora (Fig. 4D); claws blunt (Fig. 4E); gills I–VI with a membranous lamella and a fibrilliform lamella, membranous lamella of gill I leaf-like and distinctly shorter than fibrilliform lamella (Fig. 4F), gills II–VI with oval membranous lamella, fibrilliform filaments lamella of gills II–VI progressively shorter posteriorly (Fig. 4G–H); gill VII with oval membranous lamella only (Fig. 4I); supracoxal projection indistinct (Fig. 4J); posterolateral angles of terga V–IX extended into sharp projections (Fig. 4K); caudal filaments with spines on articulations (Fig. 4L).

**Figure 5.**
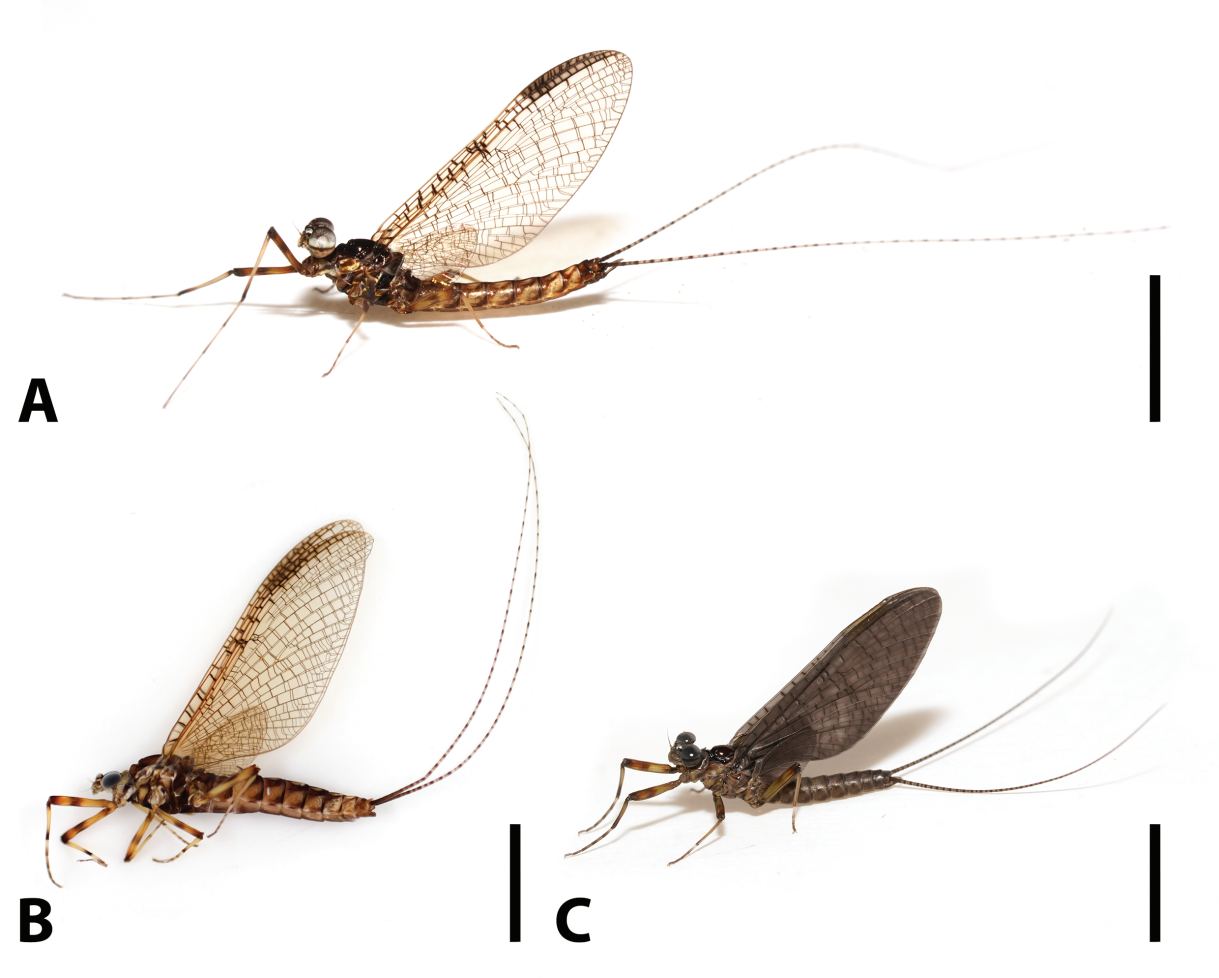
Imago of *Brachypodus quadratus* gen. et sp. nov. **A**. Male; **B**. Female; **C**. Male subimago. Scale bars: 0.5 cm (**A–C**).

**Figure 6.**
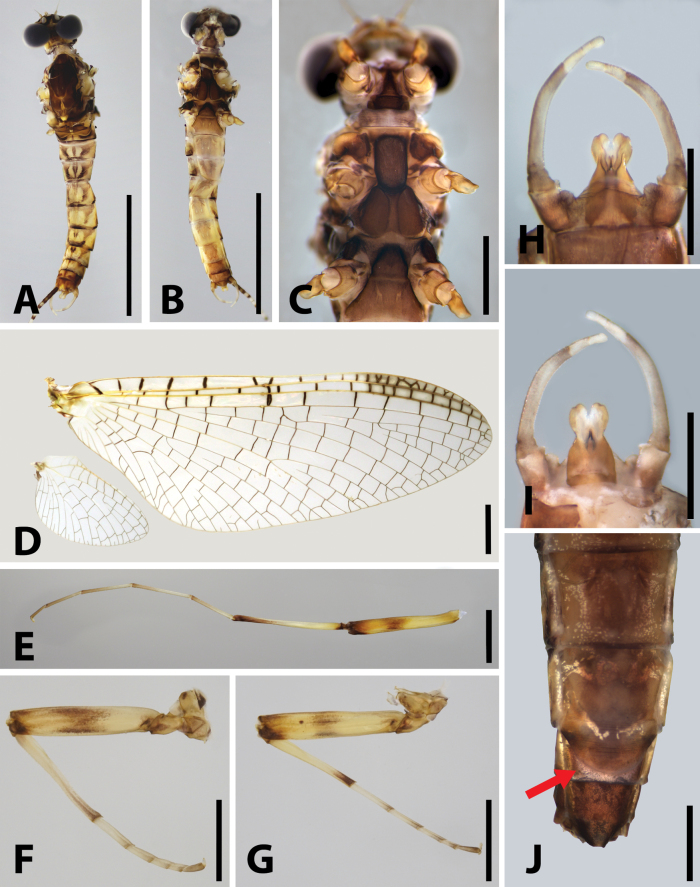
Adult structures of *Brachypodus quadratus* gen. et sp. nov. **A**. Male (dorsal view); **B**. Male (ventral view); **C**. Mesothoracic furcasternum; **D**. Forewing and hindwing of male; **E**. Foreleg of male; **F**. Midleg of male; **G**. Hindleg of male; **H**. Male genitalia (ventral view); **I**. Male genitalia (dorsal view); **J**. Terminal of female (ventral view, arrow indicating the expanded lobe of sternum VII). Scale bars: 5.0 mm (**A, B**); 1.0 mm (**C**); 0.5 mm (**D**); 5.0 mm (**E**); 1.0 mm (**G–J**).

**Imago**: body length 11.0–12.0 mm in males, slightly longer in females (Fig. 5A–C); compound eyes of male not contiguous, distance between them subequal to width of median ocellus (Fig. 6A); mesothoracic furcasternum divergent anteriorly (Fig. 6C); crossveins of wings well developed and irregularly distributed, all of them pigmented deep brown to black; 18 crossveins present between Sc and R_1,_ more than 20 between C and Sc, mostly in stigma aera (Fig. 6D); legs light brownish with dark markings (Fig. 6E–G), length of foretarsi ca 1.5× foretibiae, length of tarsomere I ca 0.85× tarsomere II in foreleg (Fig. 6E); length of hindtarsi ca 1.5× hindtibiae (Fig. 6G); two penes almost fused, with small median titillators (Fig. 6H, I); posterior margin of female sternum VII extended into semi-circular lobe (Fig. 6J).

#### Diagnosis.

*Brachypodus* gen. nov. has scattered setae on ventral surface of maxillae and three caudal filaments in nymphs, median depression of mesothoracic furcasternum with anterior divergence in adults. Those three key characters indicate it is a member of the subfamily Ecdyonurinae, which is also supported by its general morphology of nymphal mouthparts and male genitalia (Webb and McCafferty 2008).

Among genera of Ecdyonurinae, *Brachypodus* gen. nov. has at least three autapomorphies: (1) legs of nymphs short, tibiae of all legs shorter than femora, femora of fore- and midlegs strong and subquadrate (Fig. 4A–C); (2) membranous lamellae of gills I leaf-shaped and distinctly shorter than fibrilliform part (ca 0.6×), gills VII oval and without fibrilliform filament (Fig. 4F, I); (3) in male imagos, tarsomere I of foreleg ca 0.85× tarsomere II, hindtarsi longer than hindtibiae (Fig. 6E, G).

#### Comparison.

*Brachypodus* gen. nov. shares several similar characters with the genus *Thalerosphyrus* Eaton, 1881, such as laterally expanded pronotum, projected posterolateral angles of abdominal segments V–IX, and spine-whorls on caudal filaments in nymphs, relatively longer hindtarsi and fused penes in male adults. However, the two genera can be separated by supracoxal projections (invisible in *Brachypodus* gen. nov., distinct in *Thalerosphyrus*), apex of superlinguae of hypopharynx (blunt in *Brachypodus* gen. nov., acute in *Thalerosphyrus*), posterior margin of head capsule (straight in *Brachypodus* gen. nov., concave in *Thalerosphyrus*), and imaginal hindtarsi (longer than hindtibiae in *Brachypodus* gen. nov., while shorter in *Thalerosphyrus*). Their gills are also different (Eaton 1881; Webb and McCafferty 2008).

*Brachypodus* gen. nov. resembles the widespread genus *Afronurus* Lestage, 1924 in (1) mouthparts; (2) spine on caudal filaments; (3) supracoxal projections (indistinct or round). However, they can be differentiated by projected posterolateral angles of abdominal sternum V–IX (larger in *Brachypodus* gen. nov.), male penes (fused in *Brachypodus* gen. nov., while apically separated in *Afronurus*) and titillators (very small in *Brachypodus* gen. nov., distinct and separated or vestigial in *Afronurus*) (Lestage 1924; Webb and McCafferty 2008).

*Brachypodus* gen. nov. is similar to the genus *Parafronurus* in colour patterns on body and wings, shape of mouthparts, and fused penes. But the latter genus has hair-like setae on caudal filaments and shorter imaginal hindtarsi (Zhou and Braasch 2003; Webb and McCafferty 2008).

*Brachypodus* gen. nov. can be separated from *Regulaneuria* by irregular crossveins on wings (Lei et al. 2021); from *Compsoneuria* Eaton, 1881 and *Compsoneuriella* Ulmer, 1939 by having more crossveins on wings (especially in the costal regions) and the relatively longer tarsomere I of foreleg (Eaton 1881; Ulmer 1939; Webb and McCafferty 2008; Sartori 2014); from *Maculogenia* by distinctly different gills I and VII (Gong et al. 2022); from *Atopopus* Eaton, 1881 by the absence of pigments on hindwings (Eaton 1881; Webb and McCafferty 2008); from *Asionurus* Braasch & Soldán, 1986 by oval gills VII (Braasch and Soldán 1986; Webb and McCafferty 2008); from *Ecdyonurus* Eaton, 1868 by the absence of projected lobes at posterolateral angles of pronotum (Eaton 1868; Webb and McCafferty 2008).

The key differences of nymphs between the new genus *Brachypodus* gen. nov. and other related genera are listed in Table 2.

**Table 2.** Comparison between related genera of *Brachypodus* gen. nov. in Ecdyonurinae (nymph).

Genus	Anterior margin of head	Superlinguae	Gill I lamellae	Supracoxal spurs	Posterolateral spines of terga
* Brachypodus *	Not thickened	Blunt	Minute	Absent	Wide basally
* Afronurus *	Slightly thickened	Blunt	Banana shaped	Blunt	Small or narrow basally
* Asionurus *	Not thickened	Acute	Well developed	Blunt	Small or narrow basally
* Atopopus *	Distinctly thickened	Blunt	Minute	Blunt	Wide basally
* Compsoneuria *	Not thickened	Acute	Well developed	Sharp	Small or narrow basally
* Compsoneuriella *	Not thickened	Blunt	Well developed	Sharp	Small or narrow basally
* Darthus *	Slightly thickened	Blunt	Absent	Blunt	Small or narrow basally
* Ecdyonurus *	Not thickened	Blunt	Well developed	Blunt	Small or narrow basally
* Ecdyogymnurus *	Not thickened	Blunt	Well developed	Blunt	Small or narrow basally
* Maculogenia *	Slightly thickened	Blunt	Well developed	Indistinct and blunt	Small or narrow basally
* Notacanthurus *	Not thickened	Blunt	Banana shaped	Blunt	Small or narrow basally
* Parafronurus *	Slightly thickened	Blunt	Banana shaped	Blunt	Small or narrow basally
* Regulaneuria *	Not thickened	Blunt	Well developed	Blunt	Small or narrow basally
* Rhithrogeniella *	Slightly/not thickened	Blunt	Well developed	Absent/blunt	Small or narrow basally
* Thalerosphyrus *	Distinctly thickened	Acute	Well developed	Sharp	Usually large
* Thamnodontus *	Not thickened	Blunt	Well developed	Blunt	Small or narrow basally

##### Molecular distance

Based on the similarity of nymphal morphological characteristics, we chose seven genera (*Afronurus*, *Asionurus*, *Atopopus*, *Compsoneuria*, *Ecdyonurus*, *Parafronurus*, and *Thalerosphyrus*) with sequences available in GenBank for molecular analysis. Two sequences of different species were selected from each genus (Table 1). Genetic distances between these species and *Brachypodus quadratus* sp. nov. were calculated using MEGA11. The average distance between the selected species and the new species is 24.4%, with a range of 22.5% to 26.3% (Table 3). Given that the average distance amongst the chosen genera is 22.0%, the sequence of *T. vietnamensis* that is most similar to *Brachypodus quadratus* sp. nov. has a 22.5% (K2P) genetic distance from the new species.

**Table 3.** Values of K2P genetic distance among the DNA barcodes (COI).

	1	2	3	4	5	6	7	8	9	10	11	12	13
**1 *Thalerosphyrus flowersi***													
**2 *Thalerosphyrus vietnamensis***	0.239												
**3 *Parafronurus youi***	0.195	0.238											
**4 *Ecdyonurus venosus***	0.230	0.228	0.198										
**5 *Ecdyonurus insignis***	0.243	0.217	0.213	0.205									
**6 *Compsoneuria* sp. 2**	0.237	0.238	0.190	0.206	0.217								
**7 *Compsoneuria* sp. 1**	0.219	0.210	0.174	0.209	0.212	0.200							
**8 *Atopopus* sp. nov**.	0.204	0.225	0.244	0.204	0.235	0.255	0.240						
**9 *Atopopus tarsalis***	0.254	0.213	0.205	0.194	0.202	0.216	0.214	0.194					
**10 *Asionurus* sp. 1**	0.222	0.217	0.212	0.227	0.242	0.201	0.215	0.214	0.240				
**11 *Asionurus* sp. 2**	0.249	0.230	0.221	0.233	0.244	0.217	0.220	0.233	0.248	0.083			
***12 Afronurus levis***	0.221	0.231	0.191	0.255	0.237	0.210	0.200	0.191	0.196	0.189	0.192		
**13 *Afronurus* sp. nov**.	0.250	0.208	0.219	0.236	0.250	0.234	0.242	0.209	0.216	0.210	0.221	0.221	
**14 *Brachypodus quadratus***	0.247	0.225	0.237	0.237	0.256	0.243	0.247	0.250	0.236	0.247	0.249	0.263	0.230

### Brachypodus quadratus sp. nov.

Taxon classificationAnimaliaEphemeropteraHeptageniidae

E47F3899-B476-5C8B-A6A4-83B2E3D79715

https://zoobank.org/0EE34296-B721-4610-9E77-BA1ED5F89292

#### Materials examined.

***Holotype***: China • male imagos; Bajiao River, Lushui, Nujiang, Yunnan Prov; alt. 1130 m; 26°13.57'N, 99°24.31'E; 3. IV. 2025; D.W. Gong, X.H.Y. Zheng leg.; NJNUMayfly110101; ***Paratypes***: China • 26 nymphs; same data as the holotype; NJNUMayfly110102; 7 male imagos, 2 female imagos, 4 male subimagos (all adults reared from mature nymphs); same data as the holotype; NJNUMayfly110103.

#### Description.

**Mature nymph** (in alcohol, Figs 1, 2, 3, 4): body length 9.5–10.5 mm, caudal filaments 10.0–12.0 mm, body generally light yellowish, with brown stripes and spots (Fig. 1A).

***Head***: width 3.0–4.0 mm; antennae half width of head capsule; generally brown, marginal area and vertex pale; three pale patches on anterior half of head, one median, two beside compound eyes (Fig. 1C).

***Mouthparts***: labrum ca 2/3 width of anterior margin of head capsule, lateral portion extended into curved lobe, anterior margin slightly concave; both dorsal and ventral surface with dense, hair-like setae, an additional spine-like setae at median notch (Fig. 2A). Lingua bell-shaped, lateral margins of superlinguae strongly concave; apical margins of lingua and superlinguae with a row of fine, hair-like setae (Fig. 2B). Apical margins of segment II of labial palpi with dense, hair-brush; glossae and paraglossae with dense, hair-like setae on dorsal surface and apical margins; glossae subcircular; paraglossae expanded into long heart-like shape (Fig. 2C). Both mandibles with dense, hair-like setae on outer margin; prostheses consist of 7–8 bristles, upper margin near molar with a row of hair-like setae (Fig. 2D, E); both outer and inner incisors of left mandible with three apical denticles (Fig. 2D); outer incisor with three apical denticles, inner one with two of right mandible (Fig. 2E). Maxilla with 18–20 comb-like setae on crown; segment I: segment II of maxillary palpi = 1: 1.6, segment I distinctly wider than segment II; outer margins of maxillary palpi with hair-like setae, setae on segment II longer and denser than on segment I; segment II apex with a row of brush-like setae (Fig. 2F).

***Thorax***: pronotum expanding laterally, slightly wider than head capsule (Fig. 1A). Colour pattern of three legs similar: femur with one bigger median pale patch and additional three others, apex of tibia pale, apex of tarsus darker than other parts (Fig. 4A–C). Apical half of fore- and midfemora slightly expanded (Fig. 4A, B). All legs with similar setal pattern: femur with strong, hair-like setae on outer margin; tiny spatulate setae on dorsal surface (Fig. 4D); tibiae with tiny, sparse, spine-like setae and hair-like setae on anterior, posterior, and dorsal margins; tarsus with tiny, spine-like setae on inner margin and tiny, hair-like setae on surface, denser on apical margin (Fig. 4A–C); claw with three subapical denticles and a basal one (Fig. 4E). Length ratio of forefemur: tibia: tarsus ca 10.0: 9.0: 3.5 (Fig. 4A); that ratio of midleg ca 8.3: 7.3: 2.5 (Fig. 4B); ratio of hindleg ca 13.0: 11.0: 3.0 (Fig. 4C).

***Abdomen***: terga dark brown to light yellow, terga II–III, VI–VII usually darker than others; each tergum with three pairs of pale dots: submedian pair, posterior one and anterolateral one (Fig. 1A). Both dorsal and ventral lamellae of gills with clear dark tracheae; dorsal lamellae of gill I leaf-shaped, others oval (Fig. 4F–I). Terminal filament subequal to cerci, all of them with spine whorls on articulations (Fig. 4L).

**Male imago** (in alcohol, Figs 5, 6): body length 11.0–12.0 mm, forewing 11.0–12.0 mm, hindwing 2.5–3.0 mm, caudal filaments 20.0–22.0 mm. Compound eyes divided into grey upper half and black lower half.

***Thorax***: mesonotum deep reddish brown (Fig. 6A, B). Forewings transparent, only areas between C and R_1_ pale yellow with light brown stigma. C, Sc, R_1_ yellow, crossveins between them distinct, similar to crossveins between R_1_ and Rs. Other veins dark brown. Hindwings transparent, crossveins with brown pigments (Fig. 6D). Legs yellow to pale yellow; femora with medial brown band; apexes of femora, tibiae, and every tarsal segment brown (Fig. 6E–G). Length ratio of forefemur: tibia: tarsus = 1.0: 1.0: 2.0, tarsal segments from base to apex = 2.6: 3.1: 2.8: 2.0: 1.0 (Fig. 6E); length ratio of midleg is femur: tibia: tarsus = 1.5: 1.0: 1.6, length of tarsomere I > II > III > V > IV (Fig. 6F); Length ratio of hindfemur: tibia: tarsus = 3.6: 2.8: 2.9, length of tarsomere I–V ca 1: 0.7: 0.4: 0.3: 0.5 (Fig. 6G).

***Abdomen***: terga I–IX with a thin, pale, longitudinal midline; posterior margins of them with brown transverse bands. Terga pale to yellowish, with two pairs of brown to black stripes: one sub-medial straight pair and an oblique pair; tergum X generally reddish brown to black. Sterna pale but with grey to black median longitudinal stripe (Fig. 6A). Genitalia grey to brown; forceps with two pale apical segments; penes with a shallow, V-shaped medial notch (Fig. 6H, I).

**Female imago** (in alcohol, Figs 5, 6): body length 12.0–13.0 mm, forewing 12.5–13.5 mm, hindwing 4.0–4.5 mm, caudal filaments 21.5–22.5 mm. Body colour pattern similar to male, but slightly darker, abdominal markings more distinct. Forewings and hindwings semitransparent, veins clear (Fig. 5B). Length ratio of forefemur: tibia: tarsus = 1.0: 0.7: 1.0, length ratio of tarsomere I–V ca 2.0: 1.5: 1.2: 0.2: 1.0; length ratio of midfemur: tibia: tarsus = 1.3: 1.0: 1.1, length ratio of tarsomere I–V ca 1.0: 0.9: 0.7: 0.5: 0.3. Length ratio of hindfemur: tibia: tarsus = 3.8: 1.3: 1.0, length ratio of tarsomere I–V ca 1.0: 0.7: 0.5: 0.3: 0.7. Posterior margin of subgenital plate smoothly convex, posterior margin of anal plate with median notch, bottom of notch straight (Fig. 6J).

**Male subimago** (in alcohol, Fig. 5C): body length 9.5–10.5 mm, forewing 11.0–12.0 mm, hindwing 2.5–3.5 mm, caudal filaments 17.0–18.0 mm. Body brown, with indistinct patterns on abdomen terga. Length ratio of femur: tibia: tarsus ca 1.8: 1.0: 1.7 in foreleg, 1.4: 1.0: 1.1 in midleg, 1.7: 1.1: 1.0 in hindleg; length ratio of tarsomere I–V ca 1.5: 1.5: 1.5: 1.3: 1.0 in foreleg, 2.0: 1.3: 1.1: 0.7: 1.0 in midleg, hindleg tarsomere I > II > V > III > IV.

**Figure 7.**
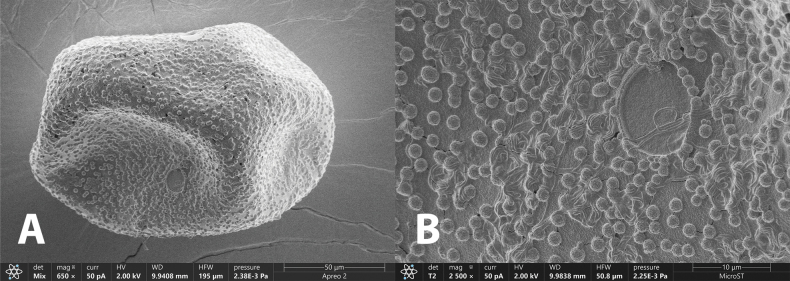
Egg of *Brachypodus quadratus* gen. et sp. nov. (SEM photo). **A**. Whole egg; **B**. Surface enlarged.

**Egg** (Fig. 7): 145.0–147.0 µm in length and 101.0–103.0 µm in width. Irregularly shaped; chorion with dense, small KCTs (knob-terminated coiled threads).

#### Biology.

**Figure 8.**
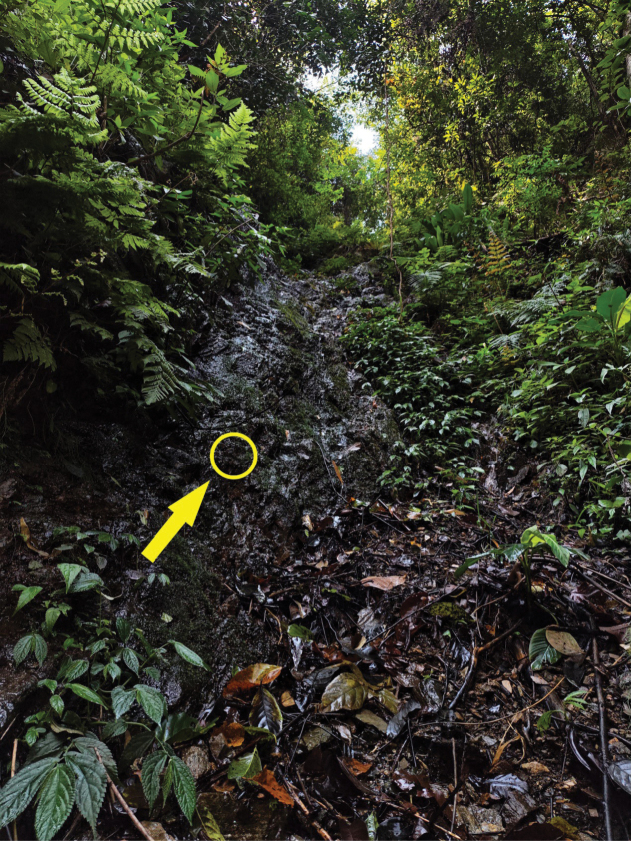
Collecting site and habitat of *Brachypodus quadratus* gen. et sp. nov. (arrow indicating the exact living point of its nymphs).

The nymphs were found on moist rock surfaces beneath a small waterfall, as well as on the damp surrounding ground (Fig. 8). The nymphs are highly agile and swiftly retreat when disturbed. We sampled several streams connected to the waterfall but did not find the species in any of them, which suggests a specific adaptation to special habitats such as thin water films and wet rock faces.

A rearing cage was positioned directly under the waterfall, maintaining conditions of high humidity and minimal water flow inside. Several mature nymphs were introduced into the cage, and subimagos emerged in the following days. The subimago stage lasted approximately 36 h.

## Discussion

The family Heptageniidae comprises approximately 37 genera and 606 species, exhibiting significantly lower generic diversity, and the definitions of its genera are often poorly defined or contentious (Jacobus et al. 2019). The subfamily Ecdyonurinae has historically been classified based on morphological phylogenies many times, resulting in several suggestions. Kluge (2004) proposed the “*Atopopus* group” based on branched maxillary dentisetae, while Wang and McCafferty (2004) established a tribal-level classification recognising multiple genera. Since then, genera in Ecdyonurinae are separated only by minute morphological differences, such as variations in dentiseta, setal patterns on the legs, and caudal filaments. However, recent molecular work has challenged former studies, highlighting the instability of current generic definitions (Yanai et al. 2017). The description and study of additional genera and species—as in the present study—will facilitate a more robust classification and a better understanding of the family’s evolution.

Although the diagnostic characters of *Brachypodus* gen. nov. are sufficient to differentiate it from other genera, its phylogenetic position remains uncertain. It exhibits a combination of characters found in several genera and is generally similar to *Thalerosphyrus* in nymphal body pattern and imaginal wings, legs and genitalia. However, it lacks supracoxal spines or acute ends of superlinguae. It also shows resemblance to *Atopopus*, but the latter genus can be differentiated by its nymphal head capsule (which has thickened anterior margin and concave posterior margin), different imaginal wings (with dark markings), and a distribution restricted to Borneo and the Philippines, rather than the Asian mainland (Eaton 1881; Webb and McCafferty 2008).

Eggs of Heptageniidae show considerable diversity, even in the same genus (see Bauernfeind and Soldán 2012). The eggs of *Brachypodus* gen. nov. are similar to those of *Thalerosphyrus* (see Boonsoong 2022), both densely covered with KCTs. Also, the eggs of *Brachypodus* gen. nov. exhibit similar surface morphology to those of *Regulaneuria cingulatus* and *Maculogenia ngi*, yet the KCTs on *Brachypodus* gen. nov. eggs are notably denser and smaller than those of the two genera (Lei et al. 2021; Gong et al. 2022).

In the field, numerous nymphs were observed with their dorsum fully exposed above the water surface, maintaining only body moisture. Several specialised morphological features appear to enable their survival and rapid movement in this unique habitat. We propose that at least the following two traits are closely linked to this adaptation: first, short and stout legs equipped with robust, curved claws. Although a variety of claw types are known in the subfamily Ecdyonurinae, the claw structure of this new genus—featuring two large denticles flanking a smaller one—is unique. A similar leg structure has evolved independently in the genus *Atopopus*, which, alongside the new genus, is the only hygropetric genus in the subfamily Ecdyonurinae (Sartori et al. 2007). Second, antenna length appears to be a significant factor. Within Ecdyonurinae, the genus *Atopopus*, which possesses the shortest antennae, is also hygropetric (Sartori et al. 2007). The discovery of this new genus, which also exhibits reduced antennae, further supports the correlation between this trait and adaptation to a hygropetric habitat.

### Key to genera in Ecdyonurinae in the Oriental Region (nymph, modified from Webb and McCafferty 2008)

**Table:** 

1	Abdominal terga with median ridge	**2**
–	Abdominal terga without median ridge	**3**
2	Lamellae of gill I absent	*** Darthus ***
–	Lamellae of gill I banana shaped	*** Notacanthurus ***
3	Apex of superlinguae distinctly pointed	**4**
–	Apex of superlinguae blunt or rounded	**6**
4	Supracoxal spurs blunt	*** Asionurus ***
–	Supracoxal spurs acutely pointed	**5**
5	Anterior margin of head capsules distinctly thickened; posterolateral spines of abdomen usually large; glossae subquadrate; posterolateral margin of head capsule often emarginate	*** Thalerosphyrus ***
–	Anterior margin of head capsules not thickened; posterolateral spines of abdomen small; glossae apically narrowed, not subquadrate; posterolateral margin of head capsule rounded	*** Compsoneuria ***
6	Posterolateral spines of abdomen wide basally	**7**
–	Posterolateral spines of abdomen small or narrow basally	**8**
7	Lamellae of gill I leaf-shaped and distinctly shorter than fibrilliform part; anterior margin of head capsule not thickened	***Brachypodus* gen. nov**.
–	Lamellae of gill I highly reduced; anterior margin of head capsule distinctly thickened	*** Atopopus ***
8	Caudal filaments with spines and whorls of long, fine setae at articulations	**9**
–	Caudal filaments with only whorls of spines, and sometimes numerous interfacing setae	**10**
9	Labrum with shallow median emargination and ventral groove, much wider than long, acuminate at both ends	*** Parafronurus ***
–	Labrum without median emargination and moderately expanded laterally, not acuminate at both ends	*** Rhithrogeniella ***
10	Hindtibiae with two dense rows of long, fine setae and usually with row of long, stout somewhat clavate setae present on lateral ridge of tibiae; head capsule slightly thickened anteriorly	***Afronurus* in part**
–	Hindtibiae with single row of long, fine setae (second, sparse row of short, fine setae with short robust setae may be present on lateral ridge) and usually without row of long stout setae; head capsule with distinct edge anteriorly and not thickened	**11**
11	Supracoxal spurs acutely pointed	*** Compsoneuriella ***
–	Supracoxal spurs indistinct or rounded	**12**
12	Terminal segment of labial palps with patch of long setae projecting medially; head capsule slightly thickened anteriorly	***Afronurus* in part**
–	Terminal segment of labial palps without patch of long setae projecting medially; head capsule not thickened anteriorly	**13**
13	Caudal filaments with numerous interfacing setae	*** Maculogenia ***
–	Caudal filaments without interfacing setae	**14**
14	Scattered setae on ventral surface of galea-laciniae fimbriate; distal dentisetae of maxillae simple or distally bifurcate	*** Ecdyogymnurus ***
–	Scattered setae on ventral surface of galea-laciniae simple; distal dentisetae of maxillae divided into several branches	**15**
15	Gill I with a concave anal margin	*** Thamnodontus ***
–	Gill I extended into projections	*** Regulaneuria ***

### Key to genera in Ecdyonurinae in the Oriental Region (male adult, modified from Webb and McCafferty 2008)

**Table:** 

1	Hindtarsi distinctly longer than hindtibiae	**2**
–	Hindtarsi shorter than or subequal to hindtibiae	**4**
2	Hindwings without any staining at margins	***Brachypodus* gen. nov**.
–	Hindwings with dark staining at margins	**3**
3	Forewing without dark staining at margins	*** Regulaneuria ***
–	Forewing with dark staining at margins	*** Atopopus ***
4	Titillators widely separated or absent	*** Afronurus ***
–	Titillators present and closely situated medially	**5**
5	Anterior margin of head distinctly produced	*** Notacanthurus ***
–	Anterior margin of head only slightly produced	**6**
6	Penes without ventral spines	**7**
–	Penes with ventral spines	**10**
7	Penes with apical sclerites distinctly separate from lateral sclerites	*** Parafronurus ***
–	Penes without apical sclerites	**8**
8	Hindtarsi less than 0.5X length of hindtibiae	*** Maculogenia ***
–	Hindtarsi greater than 0.5X length of hindtibiae	**9**
9	Forewings with red staining in C and Sc fields; penes without distinct lateral sclerites and not expanded apicolaterally	*** Asionurus ***
–	Forewings with brown or no staining in C and Sc fields; penes with distinct lateral sclerites and expanded apicolaterally	*** Thalerosphyrus ***
10	Penes with dorsolateral spines large and distinctly projected dorsally	*** Ecdyogymnurus ***
–	Penes with dorsolateral spines small and only slightly projected dorsally	*** Compsoneuria ***

## Supplementary Material

XML Treatment for Brachypodus

XML Treatment for Brachypodus quadratus

## Additional information

### Use of AI

No use of AI was reported.

### Author ORCIDs

Muhe Deng https://orcid.org/0000-0002-9640-5103

Xuhongyi Zheng https://orcid.org/0000-0002-1520-2540

Dewen Gong https://orcid.org/0000-0002-3740-7125

Changfa Zhou https://orcid.org/0000-0001-8785-5228

### Data availability

All of the data that support the findings of this study are available in the main text.

## References

[B1] Bauernfeind E, Soldán T (2012) The Mayflies of Europe (Ephemeroptera). Apollo Books, Vester Skerninge, 779 pp.

[B2] Boonsoong B (2022) Mayfly Larvae in Thailand. Pree-Wan Co., Ltd, Bangkok, 469 pp.

[B3] Braasch D, Soldán T (1986) *Asionurus* n. gen., eine neue Gattung der Heptageniidae aus Vietnam (Insecta, Ephemeroptera, Heptageniidae). Reichenbachia 23(28): 155–159.

[B4] Eaton AE (1868) Remarks upon the homologies of the ovipositor. Transactions of the Royal Entomological Society of London 16(1): 141–144. 10.1111/j.1365-2311.1868.tb00621.x

[B5] Eaton AE (1881) An announcement of new genera of the Ephemeridae. Entomologist’s Monthly Magazine 18: 21–27.

[B6] Gong DW, Zhang W, Zhou CF (2022) The real characters of *Heptagenia ngi* Hsu (1936) from China representing a new genus (Ephemeroptera: Heptageniidae). Diversity 14(12): 1027. 10.3390/d14121027

[B7] Jacobus LM, Macadam CR, Sartori M (2019) Mayflies (Ephemeroptera) and their contributions to ecosystem services. Insects 10(6): 170. 10.3390/insects10060170 PMC6628430 31207933

[B8] Kluge N (2004) The Phylogenetic System of Ephemeroptera. Kluwer Academic Publishers, Dordrecht, 442 pp. 10.1007/978-94-007-0872-3

[B9] Lei ZM, Gong DW, Zhang W, Zhou CF (2021) The first described nymphs and detailed imagoes of the species *Thalerosphyrus cingulatus* Navás revealing a new mayfly genus from eastern China (Ephemeroptera: Heptageniidae, Ecdyonurinae). Insects 12(11): 1020. 10.3390/insects12111020 PMC8618074 34821820

[B10] Lestage JA (1924) Les Ephéméres de l’Afrique du Sud. Catalogue critique & systematique des espèces connues et description de trois genera nouveaux et de sept espèces nouvelles. Revue Zoologique Africaine 12: 316–352.

[B11] Sartori M (2014) The concept of *Compsoneuria* Eaton, 1881 revisited in light of historical and new material from the Sunda Islands (Ephemeroptera, Heptageniidae, Ecdyonurinae). Zootaxa 3835(1): 1–32. 10.11646/zootaxa.3835.1.1 25081433

[B12] Sartori M, Derleth P, Webb JM (2007) The nymph of *Atopopus tarsalis* Eaton, 1881 (Ephemeroptera, Heptageniidae): First description, ecology and behavior. Zootaxa 1586(1): 25–32. 10.11646/zootaxa.1586.1.2

[B13] Ulmer G (1939) Eintagsfliegen (Ephemeropteren) von den Sunda-Inseln. Archiv für Hydrobiologie 16: 443–692.

[B14] Wang TQ, McCafferty WP (2004) Heptageniidae (Ephemeroptera) of the World. Part 1: Phylogenetic Higher Classification. Transactions of the American Entomological Society 130(1): 11–45.

[B15] Webb J, McCafferty WP (2008) Heptageniidae of the world. Part II: Key to the Genera. Canadian Journal of Arthropod Identification 7: 1–55.

[B16] Yanai Z, Sartori M, Dor R, Dorchin N (2017) Molecular phylogeny and morphological analysis resolve a long-standing controversy over generic concepts in Ecdyonurinae mayflies (Ephemeroptera: Heptageniidae). Systematic Entomology 42(1): 182–193. 10.1111/syen.12203

[B17] Zheng XHY, Zhou CF (2021) First detailed description of adults and nymph of *Cincticostella femorata* (Tshernova, 1972) (Ephemeroptera: Ephemerellidae). Aquatic Insects 42(1): 23–36. 10.1080/01650424.2020.1871026

[B18] Zhou CF, Braasch D (2003) Eine neue Gattung und Art der Heptageniidae aus dem östlichen China (Ephemeroptera). Entomologische Nachrichten und Berichte 47: 147–151.

